# Impacts of Residual Self-Interference, Hardware Impairment and Cascade Rayleigh Fading on the Performance of Full-Duplex Vehicle-to-Vehicle Relay Systems

**DOI:** 10.3390/s21165628

**Published:** 2021-08-20

**Authors:** Ba Cao Nguyen, Le The Dung, Huu Minh Nguyen, Taejoon Kim, Young-Il Kim

**Affiliations:** 1 School of Information and Communication Engineering, Chungbuk National University, Cheongju 28644, Korea; nguyenbacao@tcu.edu.vn (B.C.N.); dung.t.le@ieee.org (L.T.D.); ktjcc@chungbuk.ac.kr (T.K.); 2 Faculty of Basic Techniques, Telecommunications University, Khanh Hoa 650000, Vietnam; 3 Faculty of Radio Communications, Telecommunications University, Khanh Hoa 650000, Vietnam; nguyenhuuminh@tcu.edu.vn; 4 Electronics and Telecommunications Research Institute, Daejeon 34129, Korea

**Keywords:** full-duplex vehicle-to-vehicle communication, cascade Rayleigh fading, hardware impairment, outage probability, system throughput, ergodic capacity

## Abstract

In practice, self-interference (SI) in full-duplex (FD) wireless communication systems cannot be completely eliminated due to imperfections in different factors, such as the SI channel estimation and hardware circuits. Therefore, residual SI (RSI) always exists in FD systems. In addition, hardware impairments (HIs) cannot be avoided in FD systems due to the non-ideal characteristics of electronic components. These issues motivate us to consider an FD-HI system with a decode-and-forward (DF) relay that is applied for vehicle-to-vehicle (V2V) communication. Unlike previous works, the performance of the proposed FD-HI-V2V system is evaluated over cascaded Rayleigh fading channels (CRFCs). We mathematically obtain the exact closed-form expressions of the outage probability (OP), system throughput (ST), and ergodic capacity (EC) of the proposed FD-HI-V2V system under the joint and crossed effects of the RSI, HIs, and CRFCs. We validate all derived expressions via Monte-Carlo simulations. Based on these expressions, the OP, ST, and EC of the proposed FD-HI-V2V system are investigated and compared with other related systems, such as ideal hardware (ID) and half-duplex (HD) systems, as well as a system over traditional Rayleigh fading channels (RFCs), to clearly show the impacts of negative factors.

## 1. Introduction

Recently, vehicle-to-vehicle (V2V) communication systems have gained increasing attention thanks to their useful applications in intelligent vehicles and transport systems [1]. Simultaneously, the fast developments of autonomous vehicles in the context of Industry 4.0 and the deployment of the fifth generation (5G) of mobile networks have greatly facilitated the improvement of the quality of service (QoS) of V2V communication systems. Therefore, enhancing the QoS of V2V communication systems has become a hot topic in recent years because it can help vehicles operate autonomously without human involvement [1,2,3]. Notably, increasing the channel capacity of V2V communication systems is also a crucial requirement for vehicular networks. This is because a high channel capacity can help autonomous vehicles immediately collect data for detecting objects and connecting with other components and vehicles in safety applications. Moreover, the dense connections of the many devices in vehicles of intelligent transport systems require a substantial improvement in the spectral efficiency of V2V communication systems. This issue motivates researchers to propose various new techniques, such as full-duplex (FD), non-orthogonal multiple access (NOMA), and millimeter wave (mWave) technologies, in order to enhance the spectral efficiency of V2V wireless systems [4,5,6,7].

As demonstrated in the literature, the FD technique allows wireless devices to transmit and receive signals simultaneously on the same frequency band. This operation increases the spectral efficiency by two times compared with the traditional half-duplex (HD) mode. However, the main disadvantage of FD transmission is its induction of a strong self-interference (SI) from its output to its input [8]. Fortunately, by applying advanced SI cancellation (SIC) solutions in the antenna design, analog circuits, and digital signal processing, the SI power can be reduced to the noise floor [9,10], making FD communication feasible in practice. In addition to the benefit in capacity, the FD technique also has many other advantages, such as improving network secrecy, spectrum usage flexibility, and throughput, reducing feedback and end-to-end delays, and avoiding collision [11]. In particular, exploiting the FD technique in V2V communication systems can greatly reduce the end-to-end delay. This is because FD devices can simultaneously sense objects and send data [1,11,12].

## 2. Related Works

Many works have applied the FD technique to V2V communication systems to exploit the advantages mentioned above. Specifically, the authors in [13,14] investigated FD-V2V systems over cascaded (double) Rayleigh fading channels (CRFCs) in which the relay used FD transmission. Furthermore, both amplify-and-forward (AF) and decode-and-forward (DF) protocols at the relay were considered. Through a mathematical analysis, the authors of [13,14] derived the expressions of the outage probability (OP) and symbol error rate (SER) in FD-V2V systems. The results showed a strong impact of the residual SI (RSI) on the performance of the FD-V2V systems. In addition, the effects of CRFCs on the OP and SEP were also investigated in comparison with the traditional Rayleigh fading channel (RFC). However, the capacity of the FD-V2V system was not obtained. In [15], the authors designed a dual-band FD antenna/array for intelligent transport system applications. Their design significantly reduced the complexities of the radio-frequency front-end and the cost, weight, and size of FD antennas. To investigate the achievable rate of an FD-V2V system, the authors in [16] assumed a perfect SIC in an FD device while the interference from other vehicles affected the system. It was shown that the achievable rate of the FD-V2V system was higher than that of the HD-V2V system.

On the other hand, the design of FD radio in on-board units for V2V systems was performed in [17]. Specifically, by locating antennas separately on the rooftop of a vehicle, it was demonstrated that the isolation between the transmitting and receiving antennas of FD radios is remarkably efficient, leading to an improved SIC capability. By using separate antennas, the authors of [18,19] embedded energy harvesting (EH) for FD-V2V systems. In these works, the FD relay used AF/DF protocols and harvested the energy from the source when moving on the road. From the obtained OP and SEP expressions, the authors concluded that the performance of an FD-V2V system with EH could be optimized by using a suitable time-switching ratio that is relevant to the average transmission power of the source. In [20], FD devices were exploited in vehicular networks to enhance collision detection because FD devices can listen to channels while transmitting. Moreover, the potential of the FD technique for the enhancement of the reliability and latency when applied for automated driving purposes was also analyzed.

As discussed above, FD-V2V systems have been widely studied in terms of analyzing their performance and proposing antenna designs, as well as interference management. The effects of RSI and CRFCs have been investigated in many works. However, the case of imperfect hardware in FD-V2V systems has not been considered. Instead, most research on FD-V2V systems has evaluated the performance (OP, SEP, and EC) with ideal hardware (ID). Meanwhile, the hardware of practical devices is not perfect, especially the hardware of low-cost components, such as relays. Additionally, although different solutions have been applied to mitigate the impacts of hardware impairments (HIs), residual HIs always exist in wireless systems due to the degradation of the quality of electronic components over time [21]. Such residual HIs greatly reduce the performance of wireless communication systems, especially for systems with high data transmission rates [22,23,24,25]. Therefore, considering ideal hardware for FD-V2V systems when investigating these systems may result in an inaccurate performance evaluation.

Generally, HIs often occur at both the transmitter and receiver. Various factors cause HIs in the transceivers, such as in-phase and quadrature-phase imbalance (IQI), phase noise (PN), and the nonlinear characteristics of electronic components [21,22,23,26,27,28,29,30]. In particular, IQI refers to the phase and/or amplitude mismatch between the in-phase (I) and quadrature (Q) signals at the transmitter and receiver sides [31,32]. Specifically, the IQI is induced due to the limited accuracy of analog hardware, and it can be classified as frequency-independent or frequency-dependent. Frequency-independent IQI occurs mainly due to non-ideal mixers and phase shifters and is constant over the whole signal bandwidth, while frequency-dependent IQI is due to I and Q low-pass-filter mismatches [32]. PN is often induced by the oscillators of low-cost wireless systems because they cannot work stably with high frequencies. The nonlinear characteristics come from electronic components, such as converters (analog-to-digital and digital-to-analog), mixers, and amplifiers [21]. Different techniques have been proposed and applied in the literature to mitigate HIs in both transmitters and receivers. Specifically, various estimation and compensation approaches have been proposed to suppress the IQI and PN. In addition, the usage of highly linear components can significantly reduce the nonlinear characteristics of electronic components [21,23,27]. Although various solutions have been applied, distortion noise cannot be completely removed due to the imperfections of electronic components and parameter estimations and the random nature of hardware characteristics [21,33]. As a result, HIs still exist in wireless systems.

Motivated by these above issues, in this paper, we conduct a study of the performance of an FD-V2V system under the joint and crossed effects of RSI, HI, and CRFC with an FD-DF relay. Differing from previous works, the main contributions of our work can be summarized as follows:

1. We propose an FD-V2V system with HIs at three nodes (source, relay, and destination) in a case in which all nodes are moving vehicles (referred to as the FD-HI-V2V system from now on). The proposed FD-HI-V2V system is evaluated over cascaded Rayleigh fading channels under the joint and crossed effects of RSI, HI, and CRFC.
2. We mathematically obtain the exact closed-form expressions of the outage probability (OP), system throughput (ST), and ergodic capacity (EC) of the proposed FD-HI-V2V system with RSI and HIs over CRFCs. The derived expressions can be easily applied for related systems, such as FD-ID-V2V, HD-HI-V2V, and HD-ID-V2V systems. Then, we validate the derived expressions by using Monte-Carlo simulations.
3. We evaluate the OP, ST, and EC of the proposed FD-HI-V2V system in various scenarios and compare them with those of related systems. The numerical results demonstrate a strong effect of the RSI, HIs, and CRFCs. Specifically, the impacts of HIs and CRFCs are remarkable for high data transmission rates and high SNR regimes. Meanwhile, the effects of RSI are significant in the high SNR regime. On the other hand, when the transmission power of the source and relay is low, the performance of the proposed FD-HI-V2V system is the best when the relay is located right between the source and destination. However, when this transmission power becomes higher, the system’s performance is the best when the relay is located nearer to the source than to the destination.

The remainder of this work is organized as follows. Section 3 presents the system model of the proposed FD-HI-V2V system, and the received signals, HI, RSI, and CRFC are characterized in detail. Section 4 provides the step-by-step derivations of the exact closed-form expressions of the OP, ST, and EC of the proposed FD-HI-V2V system. Section 5 gives various numerical results and discussions. Finally, Section 6 concludes the paper.

## 3. System Model

**Figure 1:**
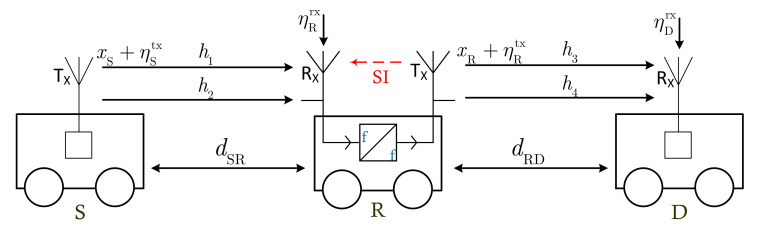
Block diagram of the proposed FD-HI-V2V system with HIs and SI.

The block diagram of the proposed FD-HI-V2V system is illustrated in Figure 1, where the source (S), relay (R), and destination (D) are moving vehicles. Specifically, S transmits signals to D via the assistance of a decode-and-forward (DF) relay. In addition, S and D use the traditional HD transmission mode, while R uses the FD transmission mode. Since the distance between S and D is great, a direct link from S to D does not exist. As a result, the proposed FD-HI-V2V system is suitable for cellular vehicle-to-everything technology, which stands for cellular communication for data transmission between vehicles.

On the other hand, because of the FD transmission mode, the input of R is affected by the SI from its output. Therefore, R should deploy all SIC solutions to suppress this SI. Furthermore, due to imperfect hardware, the signals transmitted and received at the three terminals are distorted by an HI. In particular, instead of transmitting signals $x_{S}$ and $x_{R}$ at S and R, respectively, the signals transmitted at S and R are, respectively, $x_{S}+\eta_{S}^{\mathrm{tx}}$ and $x_{R}+\eta_{R}^{\mathrm{tx}}$, where $\eta_{S}^{\mathrm{tx}}$ and $\eta_{R}^{\mathrm{tx}}$ are, respectively, the distortion noises caused by the transmitters S and R. Similarly, at the receivers, distortion noises are added to the received signals, i.e., $\eta_{R}^{\mathrm{rx}}$ and $\eta_{D}^{\mathrm{rx}}$ at R and D, respectively.

Under the effects of HIs and SI, the signal received at R is expressed as

(1) $$\begin{array}{c} y_{R}=\underset{\mathrm{desired signal and its HIs}}{\underset{︸}{\sqrt{d_{\mathrm{SR}}^{-\alpha }}h_{\mathrm{SR}}\left(x_{S}+\eta_{S}^{\mathrm{tx}}\right)+\eta_{R}^{\mathrm{rx}}}}+\underset{\mathrm{SI and its HIs}}{\underset{︸}{\sqrt{d_{\mathrm{RR}}^{-\alpha }}{\tilde{h}}_{\mathrm{RR}}\left(x_{R}+\eta_{R}^{\mathrm{tx}}\right)+{\tilde{\eta }}_{R}^{\mathrm{rx}}}}+\underset{\mathrm{Gaussian noise}}{\underset{︸}{z_{R}}}, \end{array}$$

where $d_{\mathrm{SR}}$ and $d_{\mathrm{RR}}$ denote, respectively, the distances between S and R and between the transmitting antenna and the receiving antenna of R; α∈[2,6] is the path-loss exponent; $h_{\mathrm{SR}}$ and ${\tilde{h}}_{\mathrm{RR}}$ denote the communication channel from S to R and the SI channel from the output to the input of R, respectively; $\eta_{S}^{\mathrm{tx}}∼CN\left(0,{\left(k_{S}^{\mathrm{tx}}\right)}^{2}P_{S}\right)$ and $\eta_{R}^{\mathrm{tx}}∼CN\left(0,{\left(k_{R}^{\mathrm{tx}}\right)}^{2}P_{R}\right)$ are, respectively, the distortion noises at the transmitters S and R, where CN represents a circularly symmetric complex Gaussian random variable; $k_{S}^{\mathrm{tx}}$ and $k_{R}^{\mathrm{tx}}$ denote, respectively, the HI levels at the transmitters of S and R; $P_{S}$ and $P_{R}$ are, respectively, the average transmit power at S and R; $\eta_{R}^{\mathrm{rx}}∼CN(0,{\left(k_{R}^{\mathrm{rx}}\right)}^{2}P_{S}d_{\mathrm{SR}}^{-\alpha }|h_{\mathrm{SR}}{|}^{2})$ and ${\tilde{\eta }}_{R}^{\mathrm{rx}}∼CN(0,{\left(k_{R}^{\mathrm{rx}}\right)}^{2}P_{R}|d_{\mathrm{RR}}^{-\alpha }{\tilde{h}}_{\mathrm{RR}}{|}^{2})$ are, respectively, the distortion noises caused by the receiver of R for useful (S−R) and SI (R−R) channels, where $k_{R}^{\mathrm{rx}}$ denotes the HI level at the receiver of R; $z_{R}∼CN\left(0,\sigma_{R}^{2}\right)$ is the Gaussian noise at R.

It is noted that in the proposed HI-FD-V2V system, all nodes move while they transmit/receive signals. Thus, the channels between them are characterized by cascaded Rayleigh fading [34,35]. Mathematically, the channels $h_{\mathrm{SR}}$ and $h_{\mathrm{RD}}$ are expressed as

(2) $$\begin{array}{c} h_{\mathrm{SR}}=h_{1}h_{2};\;h_{\mathrm{RD}}=h_{3}h_{4}, \end{array}$$

where $h_{1}$, $h_{2}$, $h_{3}$, and $h_{4}$ are independent Rayleigh distributions [36]. Therefore, the CDF and PDF of the cascaded channel gain $|h_{\mathrm{SR}}{|}^{2}$ are, respectively, given by [13,36]

(3) $$\begin{array}{c} F_{|h_{\mathrm{SR}}{|}^{2}}\left(x\right)=1-\sqrt{\frac{4x}{\Omega_{1}\Omega_{2}}}K_{1}\left(\sqrt{\frac{4x}{\Omega_{1}\Omega_{2}}}\right),x⩾0, \end{array}$$

(4) $$\begin{array}{c} f_{|h_{\mathrm{SR}}{|}^{2}}\left(x\right)=\frac{2}{\Omega_{1}\Omega_{2}}K_{0}\left(\sqrt{\frac{4x}{\Omega_{1}\Omega_{2}}}\right),x⩾0, \end{array}$$

where $\Omega_{1}=E\{|h_{1}{|}^{2}\}$ and $\Omega_{2}=E\{|h_{2}{|}^{2}\}$ are the average channel gains of two instantaneous channel gains, $|h_{1}{|}^{2}$ and $|h_{2}{|}^{2}$, respectively; E is the expectation operator; $K_{0}\left(.\right)$ and $K_{1}\left(.\right)$ are, respectively, the zeroth- and first-order modified Bessel functions of the second kind.

As can be seen from Equation (1), the signal received at R consists of three terms, i.e., the desired signal and its HIs, the SI and its HIs, and Gaussian noise. Since $d_{\mathrm{RR}}\ll d_{\mathrm{SR}}$, the power of the SI and its HIs is stronger than that of the desired signal. As a result, at R, it is difficult to successfully decode the received signal if SIC solutions are not applied. Therefore, in this paper, we assume that R applies all SIC solutions. First, R uses antenna placement to increase the isolation between its output and input. The usage of separate antennas for transmitting and receiving, as in this paper, can improve the SIC capability of R significantly compared with using shared antennas [10,37]. Then, analog and digital cancellations are exploited to continuously suppress the power of the SI [8,38]. By copying the output signal, R can subtract the SI signal from its input signals, especially in the digital domain [22,38]. However, R cannot suppress the SI completely due to its imperfect hardware and SI channel estimation errors. The residual SI (RSI), which is denoted by $I_{R}$, now becomes a complex Gaussian variable [9,26,38,39,40,41], i.e., $I_{R}∼CN\left(0,\sigma_{\mathrm{RSI}}^{2}\right)$, where $\sigma_{\mathrm{RSI}}^{2}=l^{2}P_{R}$, and *l* denotes the RSI level at R.

Consequently, the signal received at R becomes

(5) $$\begin{array}{cc} y_{R} & =\sqrt{d_{\mathrm{SR}}^{-\alpha }}h_{\mathrm{SR}}\left(x_{S}+\eta_{S}^{\mathrm{tx}}\right)+\eta_{R}^{\mathrm{rx}}+I_{R}+z_{R}\\ \; & =\sqrt{d_{\mathrm{SR}}^{-\alpha }}h_{\mathrm{SR}}x_{S}+\sqrt{d_{\mathrm{SR}}^{-\alpha }}h_{\mathrm{SR}}\eta_{S}^{\mathrm{tx}}+\eta_{R}^{\mathrm{rx}}+I_{R}+z_{R}. \end{array}$$

It is worth noting that improper Gaussian signals, such as RSI and HIs, can be beneficial in the presence of improper noise [42,43]. Additionally, the improper Gaussian signals can be used as an interference-management technique in some interference-limited systems. However, in this work, they are considered as undesired signals. Thus, their influences will reduce the system’s performance.

Next, R decodes the received signals, recodes them, and forwards them to D. The signal-to-interference-plus-noise-and-distortion ratio (SINDR) at R used to decode the received signal is computed as

(6) $$\begin{array}{cc} \gamma_{R} & =\frac{d_{\mathrm{SR}}^{-\alpha }{\left|h_{\mathrm{SR}}\right|}^{2}P_{S}}{d_{\mathrm{SR}}^{-\alpha }|h_{\mathrm{SR}}{|}^{2}{\left(k_{S}^{\mathrm{tx}}\right)}^{2}P_{S}+d_{\mathrm{SR}}^{-\alpha }{\left|h_{\mathrm{SR}}\right|}^{2}{\left(k_{R}^{\mathrm{rx}}\right)}^{2}P_{S}+\sigma_{\mathrm{RSI}}^{2}+\sigma_{R}^{2}}\\ \; & =\frac{|h_{\mathrm{SR}}{|}^{2}P_{S}}{|h_{\mathrm{SR}}{|}^{2}k_{\mathrm{SR}}^{2}P_{S}+d_{\mathrm{SR}}^{\alpha }\left(\sigma_{\mathrm{RSI}}^{2}+\sigma_{R}^{2}\right)}, \end{array}$$

where $k_{\mathrm{SR}}^{2}={\left(k_{S}^{\mathrm{tx}}\right)}^{2}+{\left(k_{R}^{\mathrm{rx}}\right)}^{2}$ is the aggregated HI level, which presents the HI levels at both the transmitter of S ($k_{S}^{\mathrm{tx}}$) and the receiver of R ($k_{R}^{\mathrm{rx}}$).

The signal received at D is

(7) $$\begin{array}{c} y_{D}=\sqrt{d_{\mathrm{RD}}^{-\alpha }}h_{\mathrm{RD}}\left(x_{R}+\eta_{R}^{\mathrm{tx}}\right)+\eta_{D}^{\mathrm{rx}}+z_{D}, \end{array}$$

where $d_{\mathrm{RD}}$ is the distance between R and D; $h_{\mathrm{RD}}$ denotes the communication channel from R to D; $\eta_{D}^{\mathrm{rx}}∼CN(0,{\left(k_{D}^{\mathrm{rx}}\right)}^{2}P_{R}d_{\mathrm{RD}}^{-\alpha }|h_{\mathrm{RD}}{|}^{2})$ is the distortion noise caused by the receiver of D, where $k_{D}^{\mathrm{rx}}$ represents the HI level at the receiver of D; $z_{D}∼CN\left(0,\sigma_{D}^{2}\right)$ is the Gaussian noise at D.

Using (7), the SINDR at D is computed as

(8) $$\begin{array}{c} \gamma_{D}=\frac{d_{\mathrm{RD}}^{-\alpha }{\left|h_{\mathrm{RD}}\right|}^{2}P_{R}}{d_{\mathrm{RD}}^{-\alpha }|h_{\mathrm{RD}}{|}^{2}{\left(k_{R}^{\mathrm{tx}}\right)}^{2}P_{R}+d_{\mathrm{RD}}^{-\alpha }{\left|h_{\mathrm{RD}}\right|}^{2}{\left(k_{D}^{\mathrm{rx}}\right)}^{2}P_{R}+\sigma_{D}^{2}}=\frac{|h_{\mathrm{RD}}{|}^{2}P_{R}}{|h_{\mathrm{RD}}{|}^{2}k_{\mathrm{RD}}^{2}P_{R}+d_{\mathrm{RD}}^{\alpha }\sigma_{D}^{2}}, \end{array}$$

where $k_{\mathrm{RD}}^{2}={\left(k_{R}^{\mathrm{tx}}\right)}^{2}+{\left(k_{D}^{\mathrm{rx}}\right)}^{2}$ is the aggregated HI level, which presents the HI levels at both the transmitter of R ($k_{R}^{\mathrm{tx}}$) and receiver of D ($k_{D}^{\mathrm{rx}}$).

In addition, since the DF protocol is employed at R, the end-to-end SINDR of the proposed HI-FD-V2V system is calculated as the minimum SINDR between the communication links from S to R and from R to D, i.e.,

(9) $$\begin{array}{c} \gamma_{e}2e=\min\left(\gamma_{R},\gamma_{D}\right), \end{array}$$

where $\gamma_{R}$ and $\gamma_{D}$ are calculated as in Equation (6) and Equation (8), respectively.

## 4. Performance Analysis

In this section, we first derive the OP expression and then obtain the system throughput and EC expression of the proposed HI-FD-V2V system. The detailed derivations of these expressions are provided in the following subsections.

### 4.1. Outage Probability (OP)

The OP of the proposed HI-FD-V2V system is calculated as the probability that the instantaneous data transmission rate is lower than a pre-defined value. Mathematically, the OP is given by

(10) $$\begin{array}{c} P_{\mathrm{out}}=\mathrm{Pr}\left\{\log_{2}\left(1+\gamma_{e}2e\right)<R_{0}\right\}=\mathrm{Pr}\left\{\gamma_{e}2e<2_{0}^{R}-1\right\}, \end{array}$$

where $\gamma_{e}2e$ is given as in Equation (9), and $R_{0}$ is the pre-defined data rate of the proposed system.

By setting $\gamma_{\mathrm{th}}=2_{0}^{R}-1$ as the SINDR threshold, Equation (10) now becomes

(11) $$\begin{array}{c} P_{\mathrm{out}}=\mathrm{Pr}\left\{\gamma_{e}2e<\gamma_{\mathrm{th}}\right\}. \end{array}$$

Then, the OP of the proposed HI-FD-V2V system is derived in the following Theorem 1.

**Theorem** **1.** *Under the effects of the RSI, HI, and CRFC, the OP of the proposed HI-FD-V2V system is expressed as*

(12) $$\begin{array}{c} P_{\mathrm{out}}=\left\{\begin{array}{cc} 1-\sqrt{\frac{16d_{\mathrm{SR}}^{\alpha }d_{\mathrm{RD}}^{\alpha }\left(\sigma_{\mathrm{RSI}}^{2}+\sigma_{R}^{2}\right)\sigma_{D}^{2}\gamma_{\mathrm{th}}^{2}}{\Omega_{1}\Omega_{2}\Omega_{3}\Omega_{4}P_{S}P_{R}\left(1-k_{\mathrm{SR}}^{2}\gamma_{\mathrm{th}}\right)\left(1-k_{\mathrm{RD}}^{2}\gamma_{\mathrm{th}}\right)}}K_{1}\left(\sqrt{\frac{4d_{\mathrm{SR}}^{\alpha }\left(\sigma_{\mathrm{RSI}}^{2}+\sigma_{R}^{2}\right)\gamma_{\mathrm{th}}}{\Omega_{1}\Omega_{2}P_{S}\left(1-k_{\mathrm{SR}}^{2}\gamma_{\mathrm{th}}\right)}}\right)K_{1}\left(\sqrt{\frac{4d_{\mathrm{RD}}^{\alpha }\sigma_{D}^{2}\gamma_{\mathrm{th}}}{\Omega_{3}\Omega_{4}P_{R}\left(1-k_{\mathrm{RD}}^{2}\gamma_{\mathrm{th}}\right)}}\right), & \gamma_{\mathrm{th}}<\frac{1}{\beta^{2}},\\ 1, & \gamma_{\mathrm{th}}\geq \frac{1}{\beta^{2}}, \end{array}\right. \end{array}$$

*where $\Omega_{3}=E\{|h_{3}{|}^{2}\}$ and $\Omega_{4}=E\{|h_{4}{|}^{2}\}$ are the average channel gains of R–D channel; $\beta =\max(k_{\mathrm{SR}},k_{\mathrm{RD}})$.*

**Proof**  From Equation (11), the OP can be expressed as

(13) $$\begin{array}{c} P_{\mathrm{out}}=\mathrm{Pr}\left\{\gamma_{e}2e<\gamma_{\mathrm{th}}\right\}=\mathrm{Pr}\left\{\min\left(\gamma_{R},\gamma_{D}\right)<\gamma_{\mathrm{th}}\right\}=\mathrm{Pr}\left\{\left(\gamma_{R}<\gamma_{\mathrm{th}}\right)\cup \left(\gamma_{D}<\gamma_{\mathrm{th}}\right)\right\}. \end{array}$$

Since two probabilities in Equation (13) are independent, Equation (13) can be rewritten as

(14) $$\begin{array}{c} \mathrm{Pr}\left\{\left(\gamma_{R}<\gamma_{\mathrm{th}}\right)\cup \left(\gamma_{D}<\gamma_{\mathrm{th}}\right)\right\}=\mathrm{Pr}\left\{\gamma_{R}<\gamma_{\mathrm{th}}\right\}+\mathrm{Pr}\left\{\gamma_{D}<\gamma_{\mathrm{th}}\right\}-\mathrm{Pr}\left\{\gamma_{R}<\gamma_{\mathrm{th}}\right\}\mathrm{Pr}\left\{\gamma_{D}<\gamma_{\mathrm{th}}\right\}. \end{array}$$

Therefore, we need to obtain two probabilities, e.g., $\mathrm{Pr}\{\gamma_{R}<\gamma_{\mathrm{th}}\}$ and $\mathrm{Pr}\{\gamma_{D}<\gamma_{\mathrm{th}}\}$, and then place them into Equation (14).

Then, the probability $\mathrm{Pr}\{\gamma_{R}<\gamma_{\mathrm{th}}\}$ is computed as

(15) $$\begin{array}{cc} \mathrm{Pr}\{\gamma_{R}<\gamma_{\mathrm{th}}\} & =\mathrm{Pr}\left\{\frac{|h_{\mathrm{SR}}{|}^{2}P_{S}}{|h_{\mathrm{SR}}{|}^{2}k_{\mathrm{SR}}^{2}P_{S}+d_{\mathrm{SR}}^{\alpha }\left(\sigma_{\mathrm{RSI}}^{2}+\sigma_{R}^{2}\right)}<\gamma_{\mathrm{th}}\right\}\\ \; & =\mathrm{Pr}\left\{|h_{\mathrm{SR}}{|}^{2}P_{S}\left(1-k_{\mathrm{SR}}^{2}\gamma_{\mathrm{th}}\right)<d_{\mathrm{SR}}^{\alpha }\left(\sigma_{\mathrm{RSI}}^{2}+\sigma_{R}^{2}\right)\gamma_{\mathrm{th}}\right\}. \end{array}$$

To obtain the closed-form expression from Equation (15), we must investigate two cases: $1-k_{\mathrm{SR}}^{2}\gamma_{\mathrm{th}}\leq 0$ and $1-k_{\mathrm{SR}}^{2}\gamma_{\mathrm{th}}>0$.

In the first case of $1-k_{\mathrm{SR}}^{2}\gamma_{\mathrm{th}}\leq 0$ or $\gamma_{\mathrm{th}}\geq 1/k_{\mathrm{SR}}^{2}$, we always have $\mathrm{Pr}\{\gamma_{R}<\gamma_{\mathrm{th}}\}=1$ because the left term $|h_{\mathrm{SR}}{|}^{2}P_{S}\left(1-k_{\mathrm{SR}}^{2}\gamma_{\mathrm{th}}\right)\leq 0$, while the right term $d_{\mathrm{SR}}^{\alpha }\left(\sigma_{\mathrm{RSI}}^{2}+\sigma_{R}^{2}\right)\gamma_{\mathrm{th}}>0$.

In the second case of $1-k_{\mathrm{SR}}^{2}\gamma_{\mathrm{th}}>0$ or $\gamma_{\mathrm{th}}<1/k_{\mathrm{SR}}^{2}$, Equation (15) is calculated as

(16) $$\begin{array}{cc} \mathrm{Pr}\{\gamma_{R}<\gamma_{\mathrm{th}}\} & =\mathrm{Pr}\left\{|h_{\mathrm{SR}}{|}^{2}P_{S}\left(1-k_{\mathrm{SR}}^{2}\gamma_{\mathrm{th}}\right)<d_{\mathrm{SR}}^{\alpha }\left(\sigma_{\mathrm{RSI}}^{2}+\sigma_{R}^{2}\right)\gamma_{\mathrm{th}}\right\}\\ \; & =\mathrm{Pr}\left\{|h_{\mathrm{SR}}{|}^{2}<\frac{d_{\mathrm{SR}}^{\alpha }\left(\sigma_{\mathrm{RSI}}^{2}+\sigma_{R}^{2}\right)\gamma_{\mathrm{th}}}{P_{S}\left(1-k_{\mathrm{SR}}^{2}\gamma_{\mathrm{th}}\right)}\right\}. \end{array}$$

Applying Equation (3), Equation (16) can be solved as

(17) $$\begin{array}{c} \mathrm{Pr}\left\{\gamma_{R}<\gamma_{\mathrm{th}}\right\}=1-\sqrt{\frac{4d_{\mathrm{SR}}^{\alpha }\left(\sigma_{\mathrm{RSI}}^{2}+\sigma_{R}^{2}\right)\gamma_{\mathrm{th}}}{\Omega_{1}\Omega_{2}P_{S}\left(1-k_{\mathrm{SR}}^{2}\gamma_{\mathrm{th}}\right)}}K_{1}\left(\sqrt{\frac{4d_{\mathrm{SR}}^{\alpha }\left(\sigma_{\mathrm{RSI}}^{2}+\sigma_{R}^{2}\right)\gamma_{\mathrm{th}}}{\Omega_{1}\Omega_{2}P_{S}\left(1-k_{\mathrm{SR}}^{2}\gamma_{\mathrm{th}}\right)}}\right). \end{array}$$

Combining the two cases above, the first probability $\mathrm{Pr}\{\gamma_{R}<\gamma_{\mathrm{th}}\}$ is expressed as

(18) $$\begin{array}{c} \mathrm{Pr}\left\{\gamma_{R}<\gamma_{\mathrm{th}}\right\}=\left\{\begin{array}{cc} 1-\sqrt{\frac{4d_{\mathrm{SR}}^{\alpha }\left(\sigma_{\mathrm{RSI}}^{2}+\sigma_{R}^{2}\right)\gamma_{\mathrm{th}}}{\Omega_{1}\Omega_{2}P_{S}\left(1-k_{\mathrm{SR}}^{2}\gamma_{\mathrm{th}}\right)}}K_{1}\left(\sqrt{\frac{4d_{\mathrm{SR}}^{\alpha }\left(\sigma_{\mathrm{RSI}}^{2}+\sigma_{R}^{2}\right)\gamma_{\mathrm{th}}}{\Omega_{1}\Omega_{2}P_{S}\left(1-k_{\mathrm{SR}}^{2}\gamma_{\mathrm{th}}\right)}}\right), & \gamma_{\mathrm{th}}<1/k_{\mathrm{SR}}^{2},\\ 1, & \gamma_{\mathrm{th}}\geq 1/k_{\mathrm{SR}}^{2}. \end{array}\right. \end{array}$$

Applying similar steps for calculating $\mathrm{Pr}\{\gamma_{D}<\gamma_{\mathrm{th}}\}$, we have

(19) $$\begin{array}{c} \mathrm{Pr}\left\{\gamma_{D}<\gamma_{\mathrm{th}}\right\}=\left\{\begin{array}{cc} 1-\sqrt{\frac{4d_{\mathrm{RD}}^{\alpha }\sigma_{R}^{2}\gamma_{\mathrm{th}}}{\Omega_{3}\Omega_{4}P_{R}\left(1-k_{\mathrm{RD}}^{2}\gamma_{\mathrm{th}}\right)}}K_{1}\left(\sqrt{\frac{4d_{\mathrm{RD}}^{\alpha }\sigma_{R}^{2}\gamma_{\mathrm{th}}}{\Omega_{3}\Omega_{4}P_{R}\left(1-k_{\mathrm{RD}}^{2}\gamma_{\mathrm{th}}\right)}}\right), & \gamma_{\mathrm{th}}<1/k_{\mathrm{RD}}^{2},\\ 1, & \gamma_{\mathrm{th}}\geq 1/k_{\mathrm{RD}}^{2}. \end{array}\right. \end{array}$$

Substituting Equation (18) and Equation (19) into Equation (14), we obtain the OP of the proposed HI-FD-V2V system, as shown in Theorem 1. The proof is complete. □

### 4.2. System Throughput (ST)

In addition to OP, another important parameter for analyzing the system performance is ST. Mathematically, the ST (denoted by T) is given by

(20) $$\begin{array}{c} T=R_{0}\left(1-P_{\mathrm{out}}\right), \end{array}$$

where $R_{0}$ is a pre-defined data rate and $P_{\mathrm{out}}$ is the OP of the proposed HI-FD-V2V system, which is calculated as in Equation (12).

### 4.3. Ergodic Capacity (EC)

The EC of the proposed HI-FD-V2V system can be expressed as [44]

(21) $$\begin{array}{c} E=E\left\{\log_{2}\left(1+\gamma_{e}2e\right)\right\}=\int_{0}^{\infty }\log_{2}\left(1+x\right)f_{\gamma_{e}2e}\left(x\right)dx, \end{array}$$

where $\gamma_{e}2e$ is the end-to-end SINDR of the proposed system and $f_{\gamma_{e}2e}\left(.\right)$ is the PDF of $\gamma_{e}2e$.

Applying some mathematical transforms, Equation (21) becomes

(22) $$\begin{array}{c} E=\frac{1}{\ln2}\int_{0}^{\infty }\frac{1-F_{\gamma_{e}2e}\left(x\right)}{1+x}dx, \end{array}$$

where $F_{\gamma_{e}2e}$ is the CDF of the end-to-end SINDR of the proposed HI-FD-V2V system.

From Equation (22), the EC of the proposed HI-FD-V2V system is obtained as in the following Theorem 2.

**Theorem** **2.** *The EC of the proposed HI-FD-V2V system under the effects of RSI, HI, and CRFC is given by Equation (23),*

(23) $$\begin{array}{cc} E= & \frac{\pi }{M\ln2}\sum_{m=1}^{M}\frac{\sqrt{1-\phi_{m}^{2}}}{2\beta^{2}+1+\phi_{m}}\sqrt{\frac{16d_{\mathrm{SR}}^{\alpha }d_{\mathrm{RD}}^{\alpha }\left(\sigma_{\mathrm{RSI}}^{2}+\sigma_{R}^{2}\right)\sigma_{D}^{2}}{\Omega_{1}\Omega_{2}\Omega_{3}\Omega_{4}P_{S}P_{R}}}\sqrt{\frac{{\left(1+\phi_{m}\right)}^{2}}{\left[2\beta^{2}-k_{\mathrm{SR}}^{2}\left(1+\phi_{m}\right)\right]\left[2\beta^{2}-k_{\mathrm{RD}}^{2}\left(1+\phi_{m}\right)\right]}}\\ \; & \times K_{1}\left(\sqrt{\frac{4d_{\mathrm{SR}}^{\alpha }\left(\sigma_{\mathrm{RSI}}^{2}+\sigma_{R}^{2}\right)\left(1+\phi_{m}\right)}{\Omega_{1}\Omega_{2}P_{S}\left[2\beta^{2}-k_{\mathrm{SR}}^{2}\left(1+\phi_{m}\right)\right]}}\right)K_{1}\left(\sqrt{\frac{4d_{\mathrm{RD}}^{\alpha }\sigma_{D}^{2}\left(1+\phi_{m}\right)}{\Omega_{3}\Omega_{4}P_{R}\left[2\beta^{2}-k_{\mathrm{RD}}^{2}\left(1+\phi_{m}\right)\right]}}\right), \end{array}$$

*where M is the Gauss–Chebyshev parameter; $\phi_{m}=\cos\left(\frac{(2m-1)\pi }{2M}\right)$.*

**Proof**  From the definition of $F_{\gamma_{e}2e}\left(x\right)$ [45], we can easily obtain it from the OP expression. Specifically,

(24) $$\begin{array}{cc} F_{\gamma_{e}2e}\left(x\right) & =\mathrm{Pr}\{\gamma_{e}2e<x\}\\ \; & =\left\{\begin{array}{cc} 1-\sqrt{\frac{16d_{\mathrm{SR}}^{\alpha }d_{\mathrm{RD}}^{\alpha }\left(\sigma_{\mathrm{RSI}}^{2}+\sigma_{R}^{2}\right)\sigma_{D}^{2}x^{2}}{\Omega_{1}\Omega_{2}\Omega_{3}\Omega_{4}P_{S}P_{R}\left(1-k_{\mathrm{SR}}^{2}x\right)\left(1-k_{\mathrm{RD}}^{2}x\right)}}K_{1}\left(\sqrt{\frac{4d_{\mathrm{SR}}^{\alpha }\left(\sigma_{\mathrm{RSI}}^{2}+\sigma_{R}^{2}\right)x}{\Omega_{1}\Omega_{2}P_{S}\left(1-k_{\mathrm{SR}}^{2}x\right)}}\right)K_{1}\left(\sqrt{\frac{4d_{\mathrm{RD}}^{\alpha }\sigma_{D}^{2}x}{\Omega_{3}\Omega_{4}P_{R}\left(1-k_{\mathrm{RD}}^{2}x\right)}}\right), & x<\frac{1}{\beta^{2}},\\ 1, & x\geq \frac{1}{\beta^{2}}. \end{array}\right. \end{array}$$

Substituting Equation (24) into Equation (22), we have

(25) $$E=\frac{1}{\ln2}\int_{0}^{\frac{1}{\beta^{2}}}\frac{1}{1+x}\sqrt{\frac{16d_{\mathrm{SR}}^{\alpha }d_{\mathrm{RD}}^{\alpha }\left(\sigma_{\mathrm{RSI}}^{2}+\sigma_{R}^{2}\right)\sigma_{D}^{2}x^{2}}{\Omega_{1}\Omega_{2}\Omega_{3}\Omega_{4}P_{S}P_{R}\left(1-k_{\mathrm{SR}}^{2}x\right)\left(1-k_{\mathrm{RD}}^{2}x\right)}}K_{1}\left(\sqrt{\frac{4d_{\mathrm{SR}}^{\alpha }\left(\sigma_{\mathrm{RSI}}^{2}+\sigma_{R}^{2}\right)x}{\Omega_{1}\Omega_{2}P_{S}\left(1-k_{\mathrm{SR}}^{2}x\right)}}\right)K_{1}\left(\sqrt{\frac{4d_{\mathrm{RD}}^{\alpha }\sigma_{D}^{2}x}{\Omega_{3}\Omega_{4}P_{R}\left(1-k_{\mathrm{RD}}^{2}x\right)}}\right)dx.$$

Applying ([46], Equation (25.4.30)), the integral in Equation (25) can be solved with Equation (26).

(26) $$\begin{array}{cc} \; & \int_{0}^{\frac{1}{\beta^{2}}}\frac{1}{1+x}\sqrt{\frac{16d_{\mathrm{SR}}^{\alpha }d_{\mathrm{RD}}^{\alpha }\left(\sigma_{\mathrm{RSI}}^{2}+\sigma_{R}^{2}\right)\sigma_{D}^{2}x^{2}}{\Omega_{1}\Omega_{2}\Omega_{3}\Omega_{4}P_{S}P_{R}\left(1-k_{\mathrm{SR}}^{2}x\right)\left(1-k_{\mathrm{RD}}^{2}x\right)}}K_{1}\left(\sqrt{\frac{4d_{\mathrm{SR}}^{\alpha }\left(\sigma_{\mathrm{RSI}}^{2}+\sigma_{R}^{2}\right)x}{\Omega_{1}\Omega_{2}P_{S}\left(1-k_{\mathrm{SR}}^{2}x\right)}}\right)K_{1}\left(\sqrt{\frac{4d_{\mathrm{RD}}^{\alpha }\sigma_{D}^{2}x}{\Omega_{3}\Omega_{4}P_{R}\left(1-k_{\mathrm{RD}}^{2}x\right)}}\right)dx\\ = & \frac{\pi }{2M\beta^{2}}\sum_{m=1}^{M}\frac{\sqrt{1-\phi_{m}^{2}}}{1+\frac{1}{2\beta^{2}}\left(1+\phi_{m}\right)}\sqrt{\frac{16d_{\mathrm{SR}}^{\alpha }d_{\mathrm{RD}}^{\alpha }\left(\sigma_{\mathrm{RSI}}^{2}+\sigma_{R}^{2}\right)\sigma_{D}^{2}}{\Omega_{1}\Omega_{2}\Omega_{3}\Omega_{4}P_{S}P_{R}}}\sqrt{\frac{{\left[\frac{1}{2\beta^{2}}\left(1+\phi_{m}\right)\right]}^{2}}{\left[1-\frac{k_{\mathrm{SR}}^{2}}{2\beta^{2}}\left(1+\phi_{m}\right)\right]\left[1-\frac{k_{\mathrm{RD}}^{2}}{2\beta^{2}}\left(1+\phi_{m}\right)\right]}}\\ \; & \times K_{1}\left(\sqrt{\frac{4d_{\mathrm{SR}}^{\alpha }\left(\sigma_{\mathrm{RSI}}^{2}+\sigma_{R}^{2}\right)\frac{1}{2\beta^{2}}\left(1+\phi_{m}\right)}{\Omega_{1}\Omega_{2}P_{S}\left[1-\frac{k_{\mathrm{SR}}^{2}}{2\beta^{2}}\left(1+\phi_{m}\right)\right]}}\right)K_{1}\left(\sqrt{\frac{4d_{\mathrm{RD}}^{\alpha }\sigma_{D}^{2}\frac{1}{2\beta^{2}}\left(1+\phi_{m}\right)}{\Omega_{3}\Omega_{4}P_{R}\left[1-\frac{k_{\mathrm{RD}}^{2}}{2\beta^{2}}\left(1+\phi_{m}\right)\right]}}\right). \end{array}$$

Substituting Equation (26) into Equation (25) and applying some mathematical transformations, we obtain the EC of the proposed HI-FD-V2V system as in Theorem 2. The proof is complete. □

## 5. Numerical Results and Discussions

In this section, the effects of the system parameters are evaluated by using the derived expressions of the OP, ST, and EC of the proposed FD-HI-V2V system. To indicate the impacts of the RSI, HI, and cascade Rayleigh fading channels clearly, we provide the performance of other related systems, such as the FD-ID-V2V (perfect hardware), HD-HI-V2V (perfect SIC), and FD-HI-RFC (the system over traditional Rayleigh fading channels) systems. We demonstrate the exactness of our mathematical analysis through Monte-Carlo simulation results. In all simulated scenarios, we set $P_{S}=P_{R}=P$, $\sigma_{R}^{2}=\sigma_{D}^{2}=\sigma^{2}$; the average channel gains are $\Omega_{1}=\Omega_{2}=\Omega_{3}=\Omega_{4}=1$. In the following figures, the average SNR is calculated as SNR = $P/\sigma^{2}$. In addition, the Gauss–Chebyshev parameter *M* is chosen to be M=10, which is often used in the literature, such as in [47].

**Figure 2:**
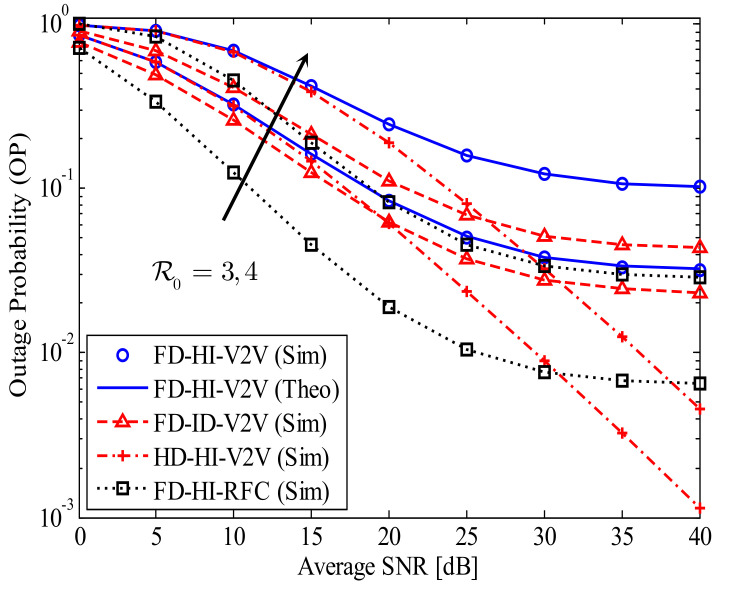
The OP of the proposed FD-HI-V2V system versus the average SNR in comparison with the OPs of the related systems; $d_{\mathrm{SR}}=d_{\mathrm{RD}}=0.5$, α=4, l=−10 dB, and $k_{S}^{\mathrm{tx}}=k_{R}^{\mathrm{tx}}=k_{R}^{\mathrm{rx}}=k_{D}^{\mathrm{rx}}=0.15$.

Figure 2 shows the OP of the proposed FD-HI-V2V system versus the average SNR in comparison with the OPs of the related systems for $d_{\mathrm{SR}}=d_{\mathrm{RD}}=0.5$, α=4, l=−10 dB, and $k_{S}^{\mathrm{tx}}=k_{R}^{\mathrm{tx}}=k_{R}^{\mathrm{rx}}=k_{D}^{\mathrm{rx}}=0.15$. The theoretical curves of the proposed FD-HI-V2V system are plotted by using Equation (12) in Theorem 1. First, it is obvious that the impact of the HI increases when the data transmission rate increases. This is because the difference between the OPs of the FD-HI-V2V system and FD-ID-V2V system in the case of $R_{0}=4$ bpcu is higher than that in the case of $R_{0}=3$ bpcu. In particular, when $R_{0}=3$ bpcu and SNR = 40 dB, the OPs are $3\times 10^{-2}$ and $2\times 10^{-2}$ for the FD-HI-V2V and FD-ID-V2V systems, respectively. As a result, the impact of the HI in this scenario is insignificant. Meanwhile, when $R_{0}=4$ bpcu and SNR = 40 dB, the OPs are $10^{-1}$ and $4\times 10^{-2}$ for the FD-HI-V2V and FD-ID-V2V systems, respectively. These OPs are significantly different, indicating that the impact of the HI in this scenario is remarkable. Secondly, the effect of the RSI due to the FD mode is very high because the OP of the FD-HI-V2V system is much higher than that of the HD-HI-V2V system. Specifically, the OP of the HD-HI-V2V system avoids the error floor, while the OP of the FD-HI-V2V system reaches the error floor in the high SNR regime. Third, the effect of the cascade Rayleigh fading channel is also significant because the OP in the case of FD-HI-RFC is much lower than the OP of the FD-HI-V2V system. The combination of the three negative factors (RSI, HI, and cascade Rayleigh fading channels) greatly increases the OP of the proposed FD-HI-V2V system. Thus, the OP of the proposed FD-HI-V2V system reaches the error floor in the high SNR regime. This result is reasonable because the SINDRs at R and D are constants; thus, the end-to-end SINDR is a constant in the high SNR regime. Specifically, in the high SNR regime, the SINDRs at R and D given in Equation (6) and Equation (8) become $\lim_{\mathrm{SNR}\to \infty }\gamma_{R}=1/\left(k_{\mathrm{SR}}^{2}+d_{\mathrm{SR}}^{\alpha }l^{2}\right)$ and $\lim_{\mathrm{SNR}\to \infty }\gamma_{D}=1/k_{\mathrm{RD}}^{2}$, respectively. Therefore, in addition to all of the SIC solutions for FD transmission, we should effectively apply various techniques to reduce the effects of HIs and cascade Rayleigh fading channels.

**Figure 3:**
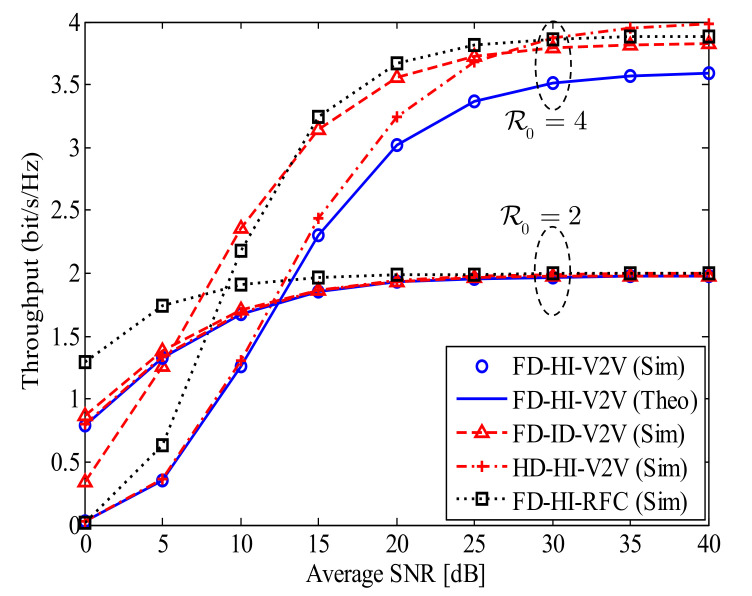
The ST of the proposed FD-HI-V2V system for two pre-defined data transmission rates; $d_{\mathrm{SR}}=d_{\mathrm{RD}}=0.5$, α=4, l=−10 dB, and $k_{S}^{\mathrm{tx}}=k_{R}^{\mathrm{tx}}=k_{R}^{\mathrm{rx}}=k_{D}^{\mathrm{rx}}=0.15$.

Figure 3 illustrates the ST of the proposed FD-HI-V2V system for two pre-defined data transmission rates, i.e., $R_{0}=2,4$ bpcu. The curves for the analysis are plotted by using Equation (20). As shown in Figure 3, for the low data transmission rate of $R_{0}=2$ bpcu, all investigated systems—FD-HI-V2V, FD-ID-V2V, HD-HI-V2V, and FD-HI-RFC—can reach the target of 2 bpcu in the high SNR regime. However, the ST of the FD-HI-RFC system is much higher than that of the other systems (FD-HI-V2V, FD-ID-V2V, and HD-HI-V2V) in the low SNR regime. This feature emphasizes the strong impact of the CRFC in comparison with the RFC in the low SNR regime. On the other hand, for the higher data transmission rate of $R_{0}=4$ bpcu, the SIs of the four systems (FD-HI-V2V, FD-ID-V2V, HD-HI-V2V, and FD-HI-RFC) are different. Specifically, the ST of the FD-ID-V2V system is the highest when SNR < 15 dB, while the ST of the HD-HI-V2V system is the highest when SNR > 30 dB. In addition, at SNR = 40 dB, the ST of the FD-HI-V2V system is 3.6 bpcu, while it is 4 bpcu for the HD-HI-V2V system. This feature demonstrates the strong impact of the RSI on the ST of the FD-HI-V2V system.

**Figure 4:**
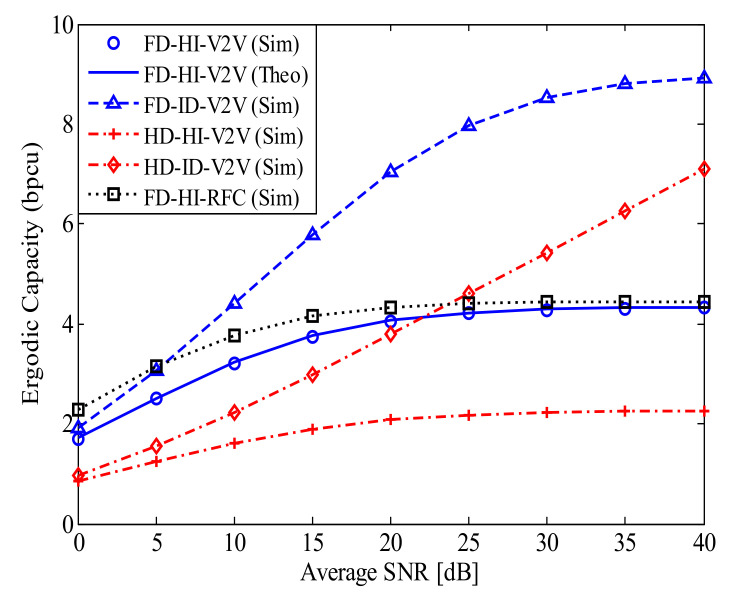
The EC of the proposed FD-HI-V2V system in comparison with the ECs of related systems; $d_{\mathrm{SR}}=d_{\mathrm{RD}}=0.5$, α=4, l=−10 dB, and $k_{S}^{\mathrm{tx}}=k_{R}^{\mathrm{tx}}=k_{R}^{\mathrm{rx}}=k_{D}^{\mathrm{rx}}=0.15$.

Figure 4 investigates the EC of the proposed FD-HI-V2V system compared to the ECs of the related systems. We use Equation (23) in Theorem 2 to plot the curves for the analysis. The system parameters used in Figure 4 are similar to those in Figure 2. As observed in Figure 4, the EC of the HD-HI-V2V system is the lowest, while the EC of the FD-ID-V2V system is the highest. In particular, with the considered RSI level of l=−10 dB, the EC of the FD-HI-V2V system is higher than that of the HD-HI-V2V system. This behavior is similar for the FD-ID-V2V and HI-ID-V2V systems. On the other hand, the EC of the FD-HI-V2V system can be higher or lower than the EC of the HD-ID-V2V system. Specifically, it is higher when SNR < 23 dB and lower when SNR > 23 dB. This feature is reasonable because a low SNR leads to low RSI power and vice versa. Furthermore, in addition to the RSI, the distortion noise power is also higher in the high SNR regime. Thus, the EC of the proposed FD-HI-V2V system reaches the capacity ceiling because the end-to-end SINDR of the proposed FD-HI-V2V system is a constant in the high SNR regime. In contrast, the HD-ID-V2V system does not suffer from both RSI and HI. Therefore, its EC still increases further with the SNR.

**Figure 5:**
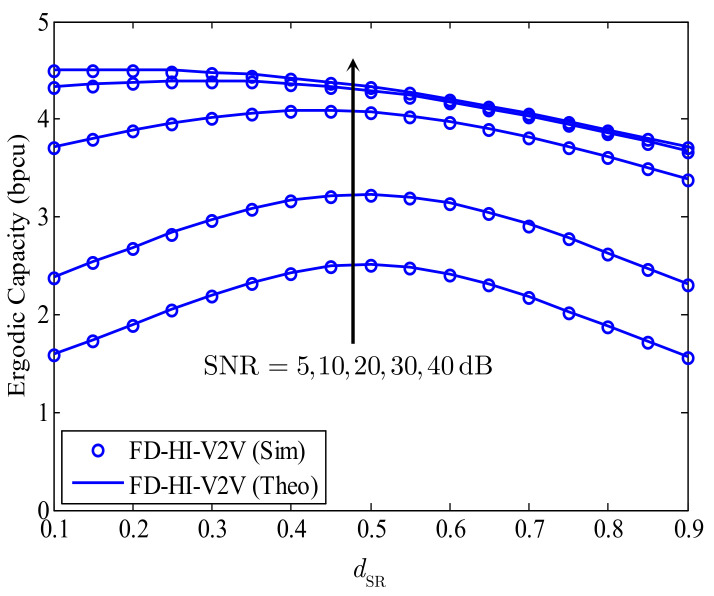
The EC of the proposed FD-HI-V2V system versus the S−R distance for different SNRs; α=4, l=−10 dB, and $k_{S}^{\mathrm{tx}}=k_{R}^{\mathrm{tx}}=k_{R}^{\mathrm{rx}}=k_{D}^{\mathrm{rx}}=0.15$.

Figure 5 shows the EC of the proposed FD-HI-V2V system versus the S–R distance for different SNRs with $d_{\mathrm{SR}}+d_{\mathrm{RD}}=1$. We can see that, when the transmission power of S and R is low, i.e., SNR = 5, 10, 20 dB, the EC of the proposed system is maximal when $d_{\mathrm{SR}}=d_{\mathrm{RD}}$. However, when the transmission power of S and R increases, i.e., SNR = 30 or 40 dB, the EC is maximal when the relay is nearer to the source than to the destination. For example, with SNR = 30 dB, the EC is maximal when $d_{\mathrm{SR}}=0.4$ and $d_{\mathrm{RD}}=0.6$. This feature is reasonable for the proposed FD-HI-V2V system because the EC of the proposed system gets higher when both $\gamma_{R}$ and $\gamma_{D}$ increase according to Equation (9). For the investigated parameters, the average channel gains of the S−R and R−D links are identical. Thus, when the SNR increases, $\gamma_{D}$ rapidly increases, while $\gamma_{R}$ increases slowly due to the effect of the RSI. Therefore, the S−R distance must be reduced to increase $\gamma_{R}$, as presented in Equation (6). As a result, depending on the average SNR, we can choose a suitable location of R to enhance the EC of the proposed FD-HI-V2V system.

**Figure 6:**
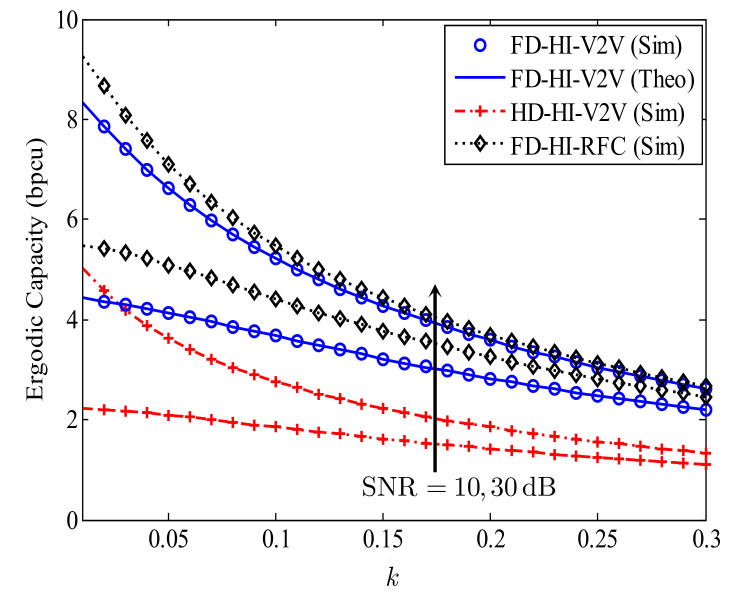
The effect of distortion noise *k* on the EC of the proposed FD-HI-V2V system; $d_{\mathrm{SR}}=d_{\mathrm{RD}}=0.5$, α=4, and l=−10 dB.

Figure 6 investigates the effect of distortion noise *k* on the EC of the proposed FD-HI-V2V system, where $k_{S}^{\mathrm{tx}}=k_{R}^{\mathrm{tx}}=k_{R}^{\mathrm{rx}}=k_{D}^{\mathrm{rx}}=k$. As shown in Figure 6, the effect of the CRFC is remarkable when the level of distortion noise is low, as illustrated through the significant difference between the ECs of the FD-HI-V2V and FD-HI-RFC systems for small values of *k*. In particular, with SNR = 10 dB and k=0, the EC of the FD-HI-RFC system is 1 bpcu higher than the EC of the proposed FD-HI-V2V system. When *k* increases, the impact of the CRFC decreases. Specifically, when k=0.3, the ECs of both the FD-HI-V2V and FD-HI-RFC systems are almost equal. Furthermore, when *k* increases from 0 to 0.3, the EC of the proposed FD-HI-V2V system is reduced from 4.2 to 2.1 and 8.1 to 2.3 bpcu for SNR = 10 and SNR = 30 dB, respectively. This result clearly indicates the effect of the HI on the EC of the proposed system. On the other hand, the EC of the proposed FD-HI-V2V system is always higher than the EC of the HD-HI-V2V system. This characteristic demonstrates the benefit of the FD transmission mode compared with the traditional HD transmission mode in terms of the EC.

**Figure 7:**
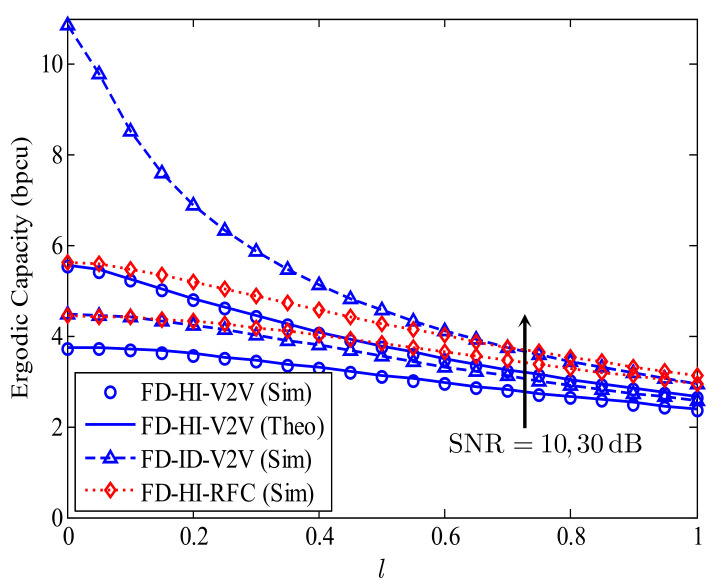
The impact of the RSI due to the FD transmission mode on the EC of the proposed FD-HI-V2V system; $k_{S}^{\mathrm{tx}}=k_{R}^{\mathrm{tx}}=k_{R}^{\mathrm{rx}}=k_{D}^{\mathrm{rx}}=0.1$.

In addition to the HI and CRFC, the RSI due to the FD transmission mode also greatly influences the EC of the proposed FD-HI-V2V system. Thus, in Figure 7, we investigate the EC versus the RSI level *l* for two SNR settings, i.e., SNR = 10 and 30 dB. Notice that in the case of l=0, the EC of the proposed FD-HI-V2V system becomes the EC of the HD-HI-V2V system. Similarly to the HI, the RSI significantly reduces the EC of the proposed FD-HI-V2V system. As can be seen from Figure 7, when the RSI level *l* increases from 0 (perfect SIC) to 1 (without SIC), the ECs are greatly reduced, especially for the FD-ID-V2V system. In particular, the EC of the FD-ID-V2V system is reduced from 11 to 3 bpcu for the investigated range of *l*. For the proposed FD-HI-V2V system, the EC is only 5.5 bpcu when l=0 and SNR = 30 dB due to the HI. In addition, the effect of the CRFC is more substantial for higher values of *l*.

## 6. Conclusions

In the literature, the CRFC has been proven as an excellent model for describing V2V communication channels in practice. However, it is usually neglected when mathematically analyzing V2V communication systems because of its complexity. Additionally, the HI and RSI caused by imperfect hardware and SIC always affect FD communication systems. In this paper, we investigated the performance of an FD-HI-V2V system in terms of OP, ST, and EC under the impacts of HI, RSI, and CRFC. We first derived the expressions of the OP, ST, and EC of the proposed FD-HI-V2V system and then used the obtained expressions to evaluate the system’s performance. The numerical results show a severe degradation in the performance of the proposed system because of the HI, RSI, and CRFC in comparison with related systems, such as the FD-ID-V2V, HD-HI-V2V, FD-HI-RFC, HI-ID-V2V systems. Specifically, under the impacts of HI, RSI, and CRFC, the OP, ST, and EC of the proposed FD-HI-V2V system reach saturated values in the high SNR regime. In particular, the OP goes to the saturated minimum value quickly for high data transmission rates, even when the HI and RSI levels are small. Meanwhile, the ST and EC reach saturated maximum values for SNR ≥ 30 dB. Thus, we should use low data transmission rates and transmission power for FD-HI-V2V systems. In addition, when the transmission power is high, putting the relay nearer to the source than to the destination provides a higher system performance. More importantly, to enhance the performance of FD-HI-V2V systems, wireless designers and researchers need to put in more effort to suppress HIs and RSI before deploying them in practical scenarios. Therefore, studying various methods to reduce the HI and RSI in order to improve the performance of FD-HI-V2V systems and considering the case of multiple users are the objectives of our future work. 

## Data Availability

The data presented in this study are available on request from the corresponding author. The data are not publicly available due to privacy restrictions.
